# Supplementary material for: A corrosion-resistant RuMoNi catalyst for efficient and long-lasting seawater oxidation and anion exchange membrane electrolyzer
Source: Nat Commun. 2023 Jun 17;14:3607. doi: 10.1038/s41467-023-39386-5 (PMC10276855; doi:10.1038/s41467-023-39386-5)
Supplement: Supplementary file 1 — Supplementary Information [file 41467_2023_39386_MOESM1_ESM.pdf]

Supplementary Information

A corrosion-resistant RuMoNi catalyst for efficient and long-lasting seawater oxidation and anion exchange membrane electrolyzer

Xin Kang[1], Fengning Yang[1,2], Zhiyuan Zhang[1], Heming Liu[1], Shiyu Ge[1], Shuqi Hu[1], Shaohai Li[1], Yuting Luo[1,3], Qiangmin Yu[1*], Zhibo Liu[4], Qiang Wang[4], Wencai Ren[4], Chenghua Sun[5], Hui-Ming Cheng[4,6,7], and Bilu Liu[1*]

1. Shenzhen Geim Graphene Center, Tsinghua-Berkeley Shenzhen Institute & Institute of Materials Research, Tsinghua Shenzhen International Graduate School, Tsinghua University, Shenzhen 518055, P.R. China
2. Department of Physics, University of Oxford, Clarendon Laboratory, Parks Road, Oxford, OX1 3PU, UK
3. Department of Electrical and Computer Engineering, University of Toronto, 35 St George Street, Toronto, Ontario M5S 1A4, Canada.
4. Shenyang National Laboratory for Materials Science, Institute of Metal Research, Chinese Academy of Sciences, Shenyang 110016, P.R. China
5. Department of Chemistry and Biotechnology, Swinburne University of Technology, Hawthorn, VIC, 3122, Australia
6. Faculty of Materials Science and Engineering, Institute of Technology for Carbon Neutrality, Shenzhen Institute of Advanced Technology, Chinese Academy of Sciences, Shenzhen 518055, P.R. China
7. Advanced Technology Institute, University of Surrey, Guildford, GU2 7XH, UK

Corresponding authors

Bilu Liu, email: bilu.liu@sz.tsinghua.edu.cn

Qiangmin Yu, email: yu.qiangmin@sz.tsinghua.edu.cn

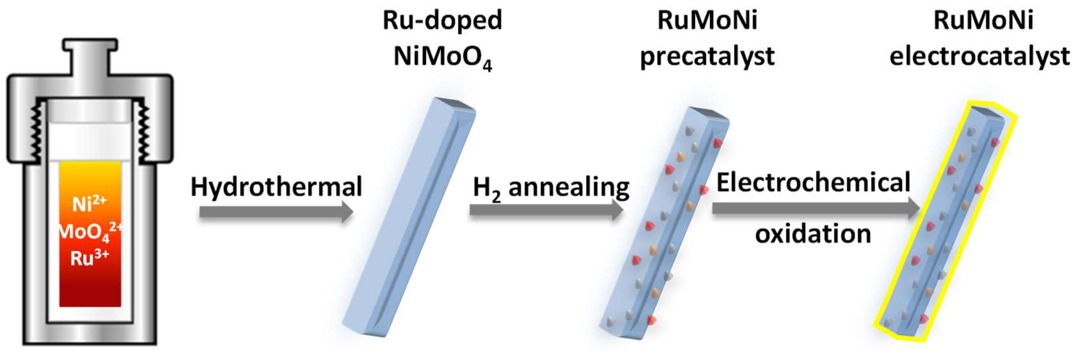

Supplementary Figure 1. Schematic of the preparation of the RuMoNi electrocatalyst. The red, orange and dark grey dots represent active sites distributed on the surface of the nanorod, and the yellow outline represents the anti-corrosion layer.

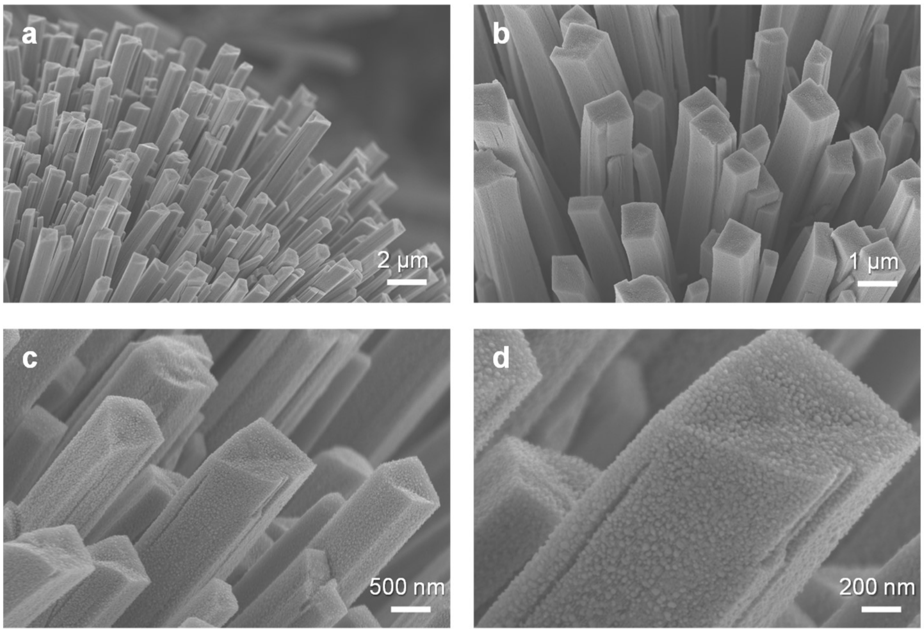

Supplementary Figure 2. SEM images of the RuMoNi precatalyst.

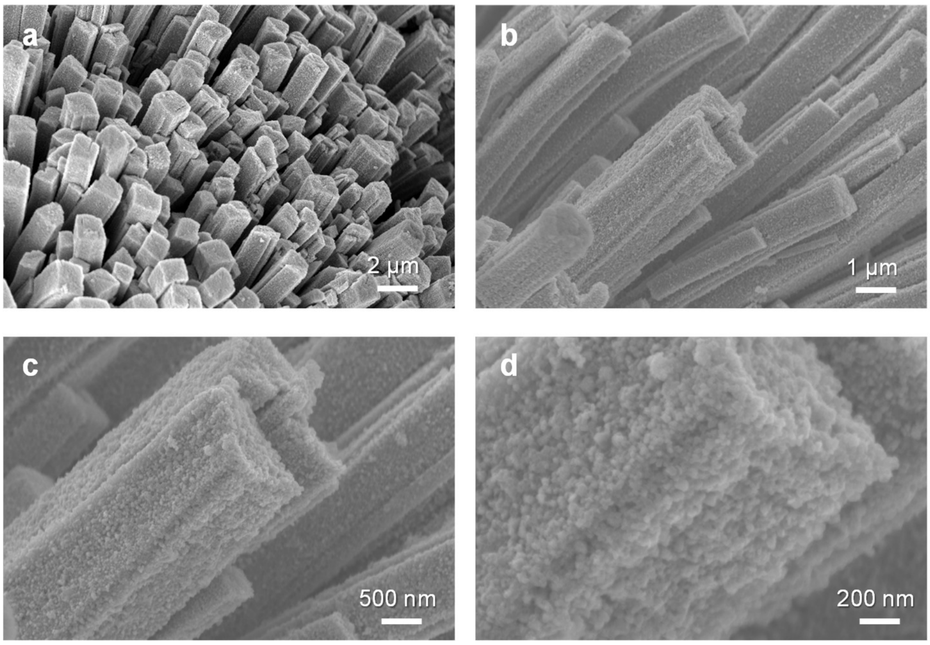

Supplementary Figure 3. SEM images of the RuMoNi electrocatalyst.

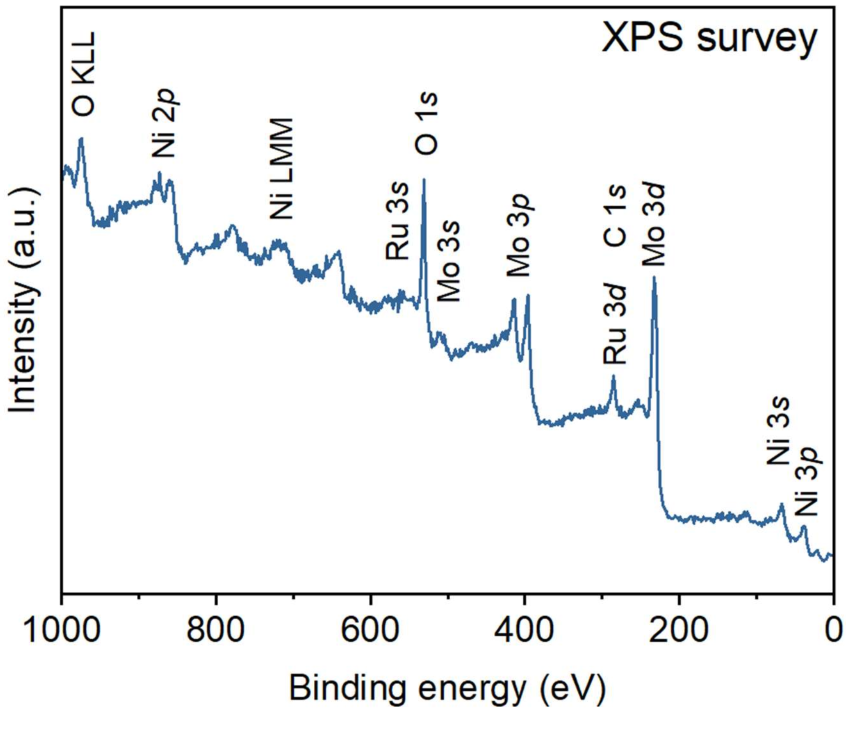

Supplementary Figure 4. XPS survey result of the RuMoNi electrocatalyst. The unit of the intensity is arbitrary units (a.u.).

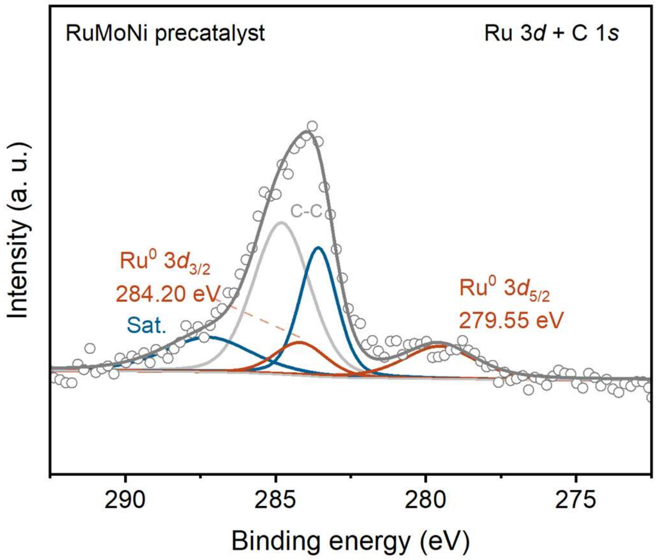

Supplementary Figure 5. The XPS spectrum of Ru 3*d* in RuMoNi precatalyst. Ru shows a peak at 279.55 eV corresponding to the binding energy of Ru^0 3$d_{5/2}$[1], which indicates the existence of metallic Ru in the precatalyst.

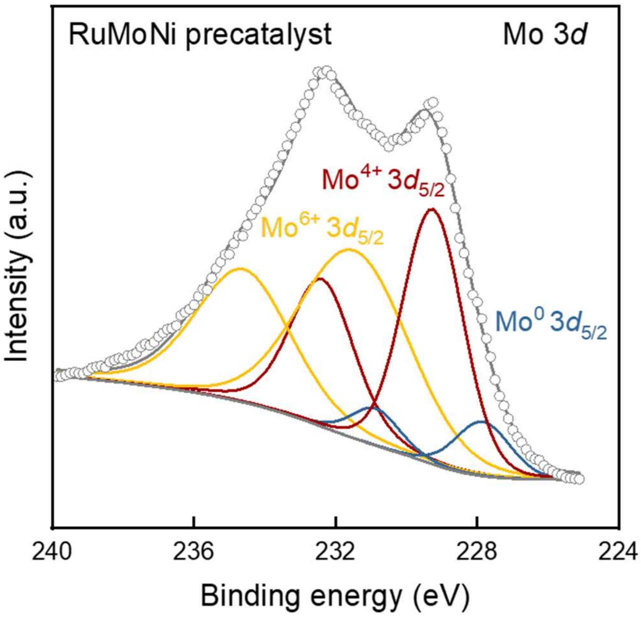

Supplementary Figure 6. The XPS spectrum of Mo 3*d* in RuMoNi precatalyst. In this figure, we can find doublets of Mo^0, Mo^{4+}, and Mo^{6+} with 3$d_{5/2}$ bands centered at 227.84 eV, 229.25 eV, and 231.47 eV, respectively[2-4], which confirm the metallic state of Mo and suggest the coexistence of molybdenum oxides in the precatalyst.

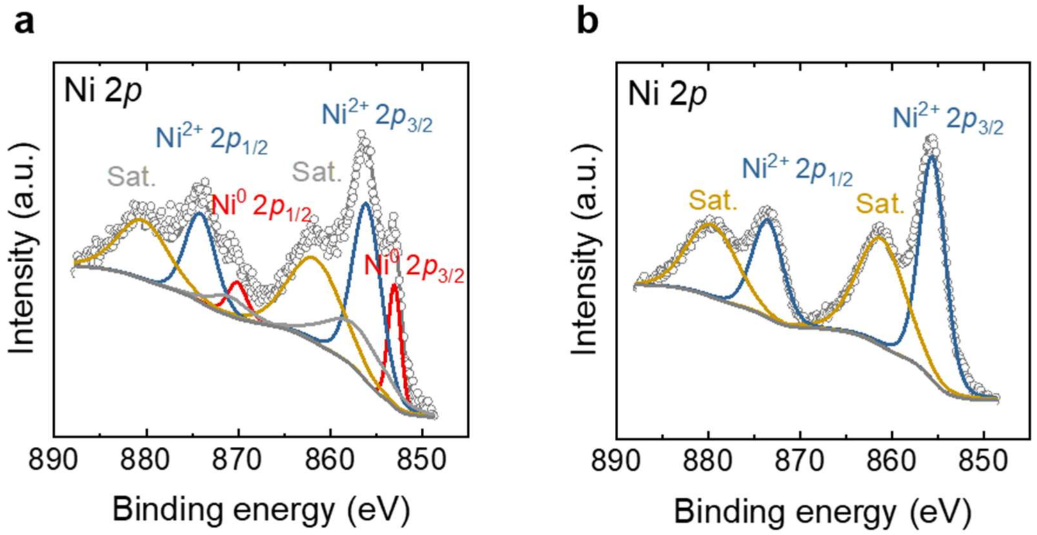

Supplementary Figure 7. Ni 2*p* XPS spectra of the RuMoNi. **a)** Ni 2*p* spectrum of the RuMoNi precatalyst shows peaks of Ni^0 and Ni^{2+}. **b)** Ni 2*p* spectrum of RuMoNi catalyst shows a doublet of Ni^{2+}. These results indicate the oxidation of Ni atoms on the precatalyst surface during electrochemical oxidation.

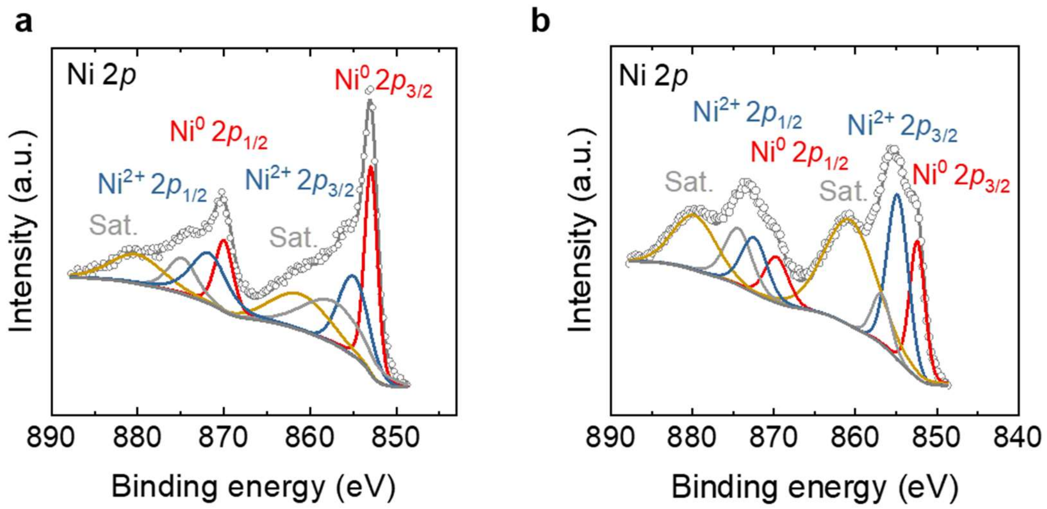

Supplementary Figure 8. Ni 2*p* XPS spectra of the RuMoNi after Ar^+ ion etching. **a)** Ni 2*p* spectrum of the RuMoNi precatalyst. **b)** Ni 2*p* spectrum of the RuMoNi catalyst after 100 h electrolysis. The Ni^0 peak indicates the stable existence of Ni_4Mo inside the nanorod during the electrochemical reaction.

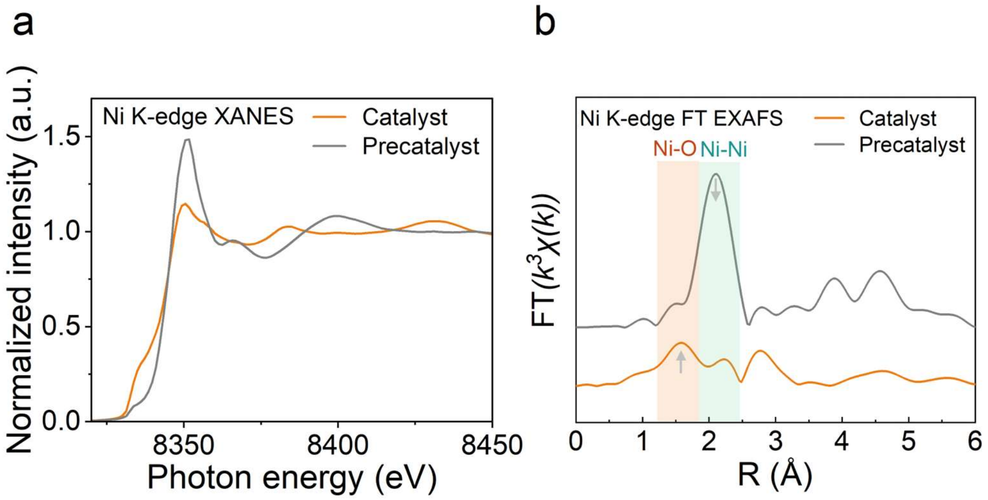

Supplementary Figure 9. XAFS and FT-EXAFS spectra of the RuMoNi precatalyst and RuMoNi catalyst to study Ni valance transformation during the electrochemical oxidation. **a)** XAFS spectra of the RuMoNi precatalyst and RuMoNi catalyst at the Ni K-edge. **b)** FT-EXAFS spectra of the RuMoNi precatalyst and RuMoNi catalyst in a 1.0 M KOH + 0.5 M KCl electrolyte. These results indicated that Ni went through a quick oxidation.

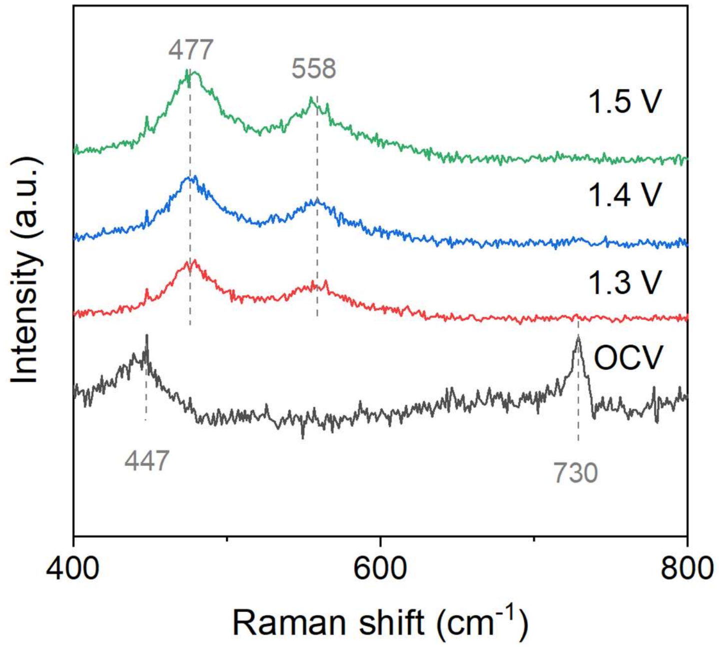

Supplementary Figure 10. In situ Raman spectra of the RuMoNi precatalyst at different applied potentials. The band at 447 cm^{-1} is the characteristic peak of $Ni(OH)_2$[5,6]. The band at 730 cm^{-1} is the anti-symmetric vibration of O-Mo-O[7,8]. Under the applied voltage of 1.3 V, 1.4 V, and 1.5 V, bands at 477 cm^{-1} and 558 cm^{-1} are the characteristic peaks of NiOOH[6,9]. This result indicates that the reconstructed area consists of NiOOH after electrochemical activation. However, no related signal of NiOOH can be found in XRD (Fig. 4e). For the amorphous phase on the surface, the above results indicate that it contains NiOOH.

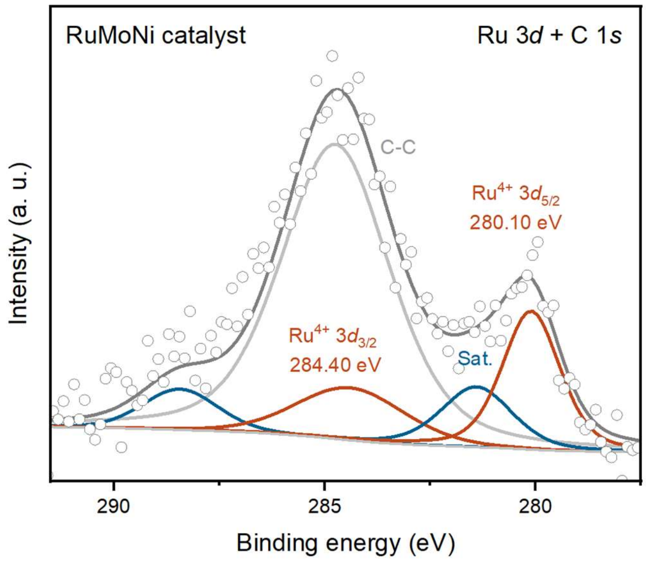

Supplementary Figure 11. XPS spectrum of Ru 3*d*. This result shows that Ru exists as Ru^{4+} during OER.

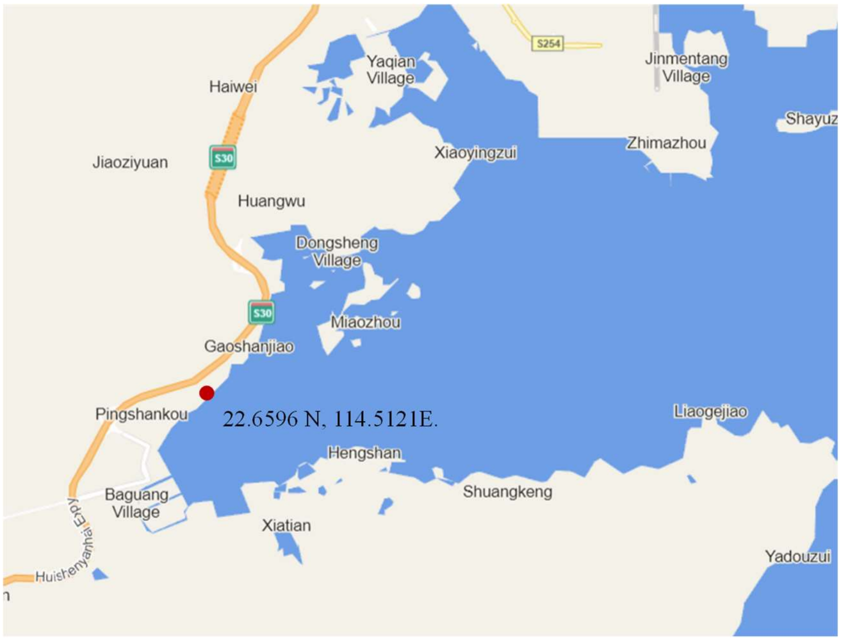

Supplementary Figure 12. Location of seawater sampling in 21/11/2021. The raw seawater was obtained from Huizhou, Guangdong province, China. 22.6596 N, 114.5121E.

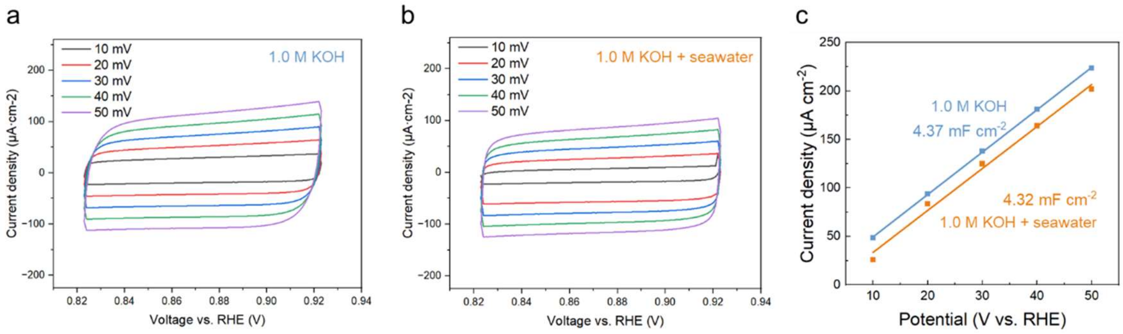

Supplementary Figure 13. CV curves of RuMoNi in a) 1.0 M KOH, b) 1.0 M KOH + seawater at 20 ± 2 °C. c) Capacitive currents at 0.87 V vs. RHE against scan rates for RuMoNi in 1.0 M KOH and 1.0 M KOH + seawater. The R^2 values for the lines of RuMoNi in 1.0 M KOH and 1.0 M KOH + seawater are 0.999, and 0.992, respectively.

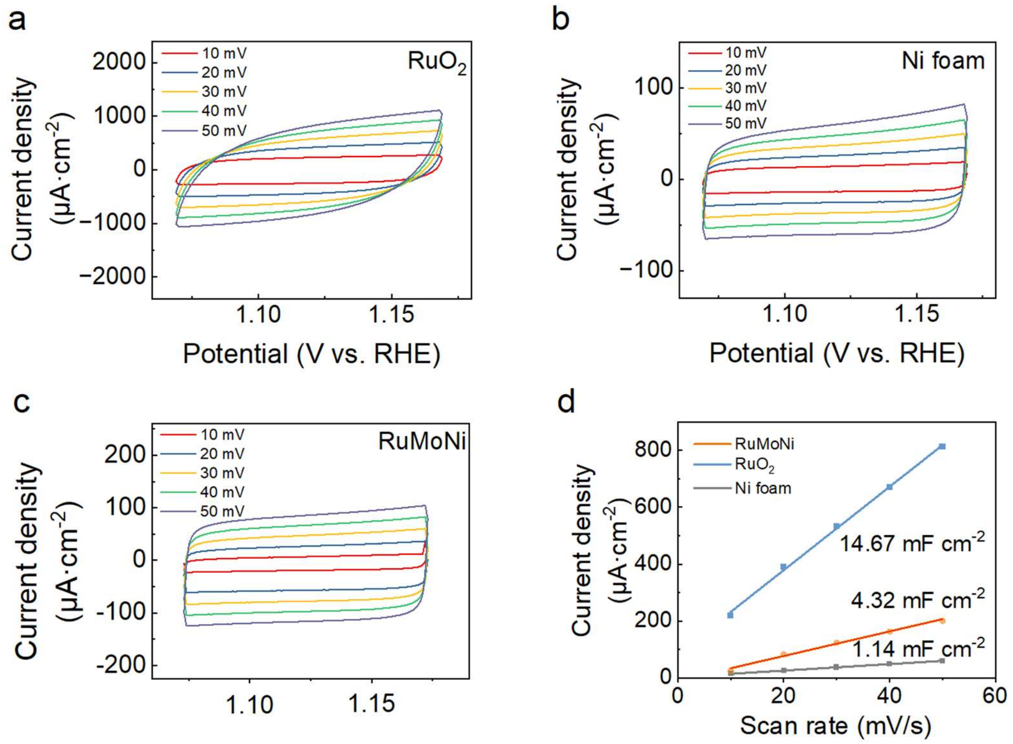

Supplementary Figure 14. CV curves of **a)** RuO_2, **b)** Ni foam, and **c)** RuMoNi. **d)** Capacitive currents at 1.125 V vs. RHE against scan rate for RuMoNi, RuO_2, and Ni foam at 20 ± 2 °C. The R^2 values for the lines of RuMoNi, RuO_2 and Ni foam are 0.992, 0.998, and 0.999, respectively.

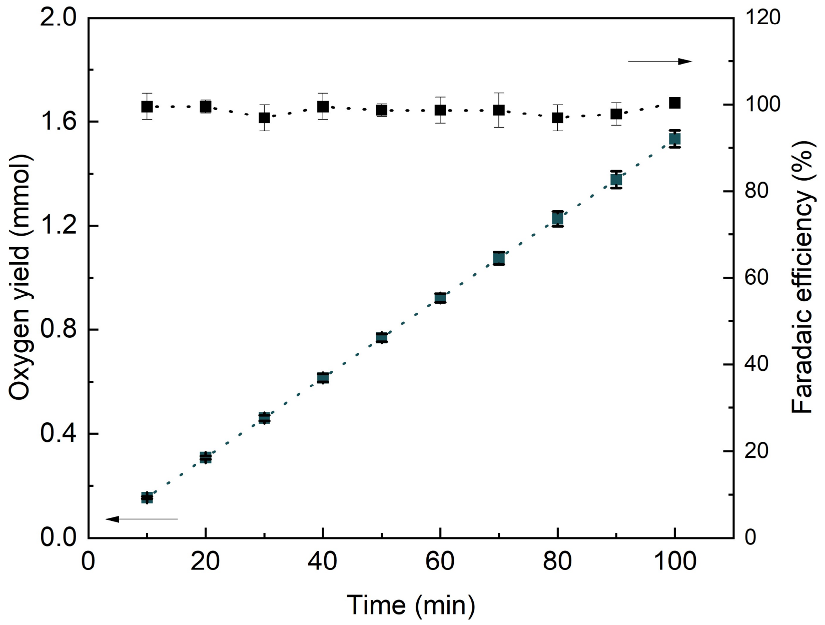

Supplementary Figure 15. Faradaic efficiency of OER using a drainage gas collection method at 20 ± 2 °C, which shows that the OER selectivity of the RuMoNi is ~100%. The error bars correspond to standard deviations of the data from three experiments.

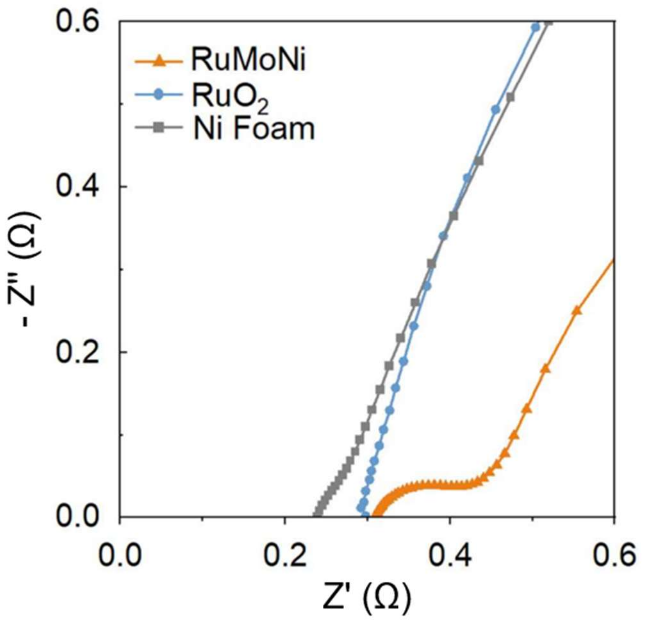

Supplementary Figure 16. Enlarged electrochemical impedance spectra of RuMoNi, RuO_2, and Ni Foam at 20 ± 2 °C with symbols of raw data and lines of fitting data.

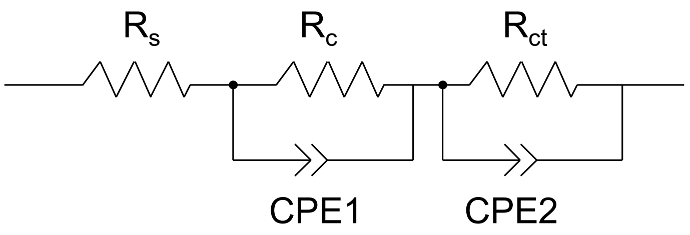

Supplementary Figure 17. The equivalent circuit for impedance spectra data of three catalysts. (Note: the fitting software is Z-view.). R_s is the resistance of the solution, R_c is the resistance of the corrosion layer and R_{ct} is the charge transfer resistance on the electrode surface. CPE1 and CPE2 stand for the constant phase elements[10].

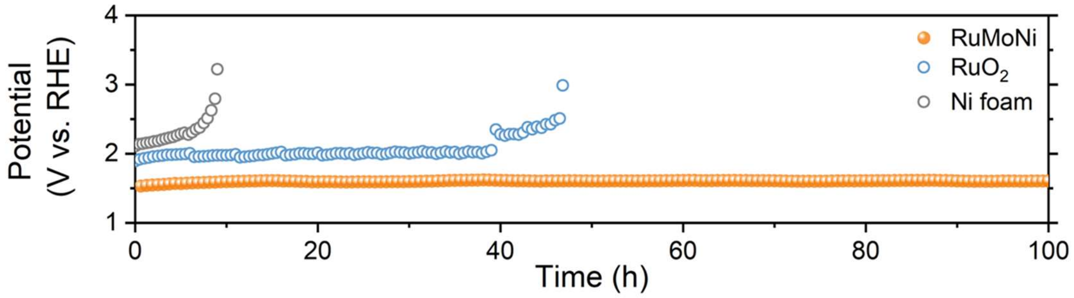

Supplementary Figure 18. Durability test for the OER of RuO_2, Ni foam, and RuMoNi recorded at a current density of 500 mA cm^{-2} of the seawater electrolysis in a 1.0 M KOH + seawater electrolyte at 20 ± 2 °C.

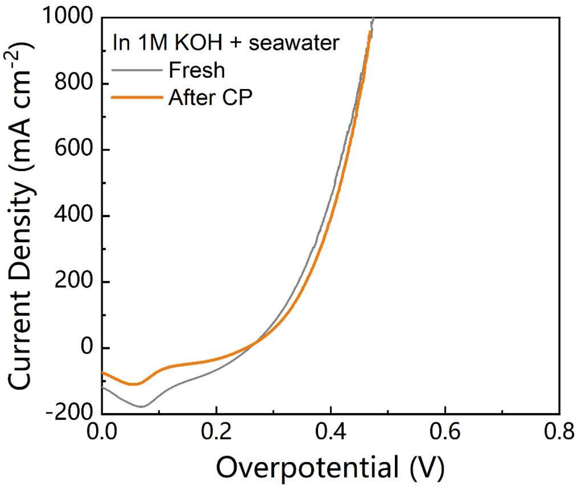

Supplementary Figure 19. LSV curves of the RuMoNi catalyst before and after a 3,000 h CP test at 20 ± 2 °C. The negligible difference between the curves shows the high durability of the RuMoNi.

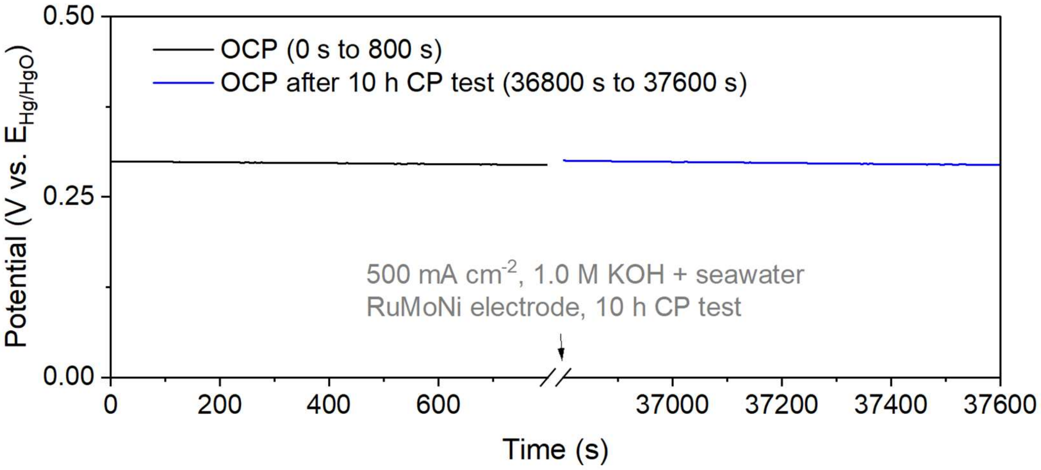

Supplementary Figure 20. Open circuit potential (OCP) of RuMoNi before and after 10 h stability test in 1.0 M KOH + seawater at a current density of 500 mA cm^{-2} at 20 ± 2 ℃.

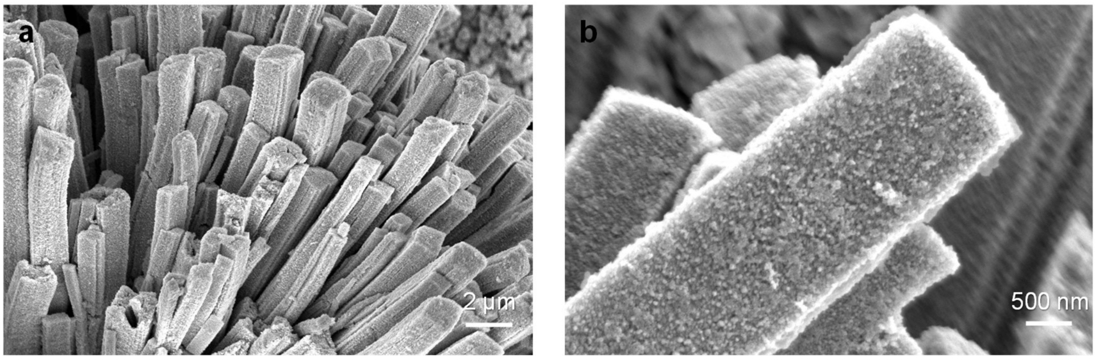

Supplementary Figure 21. SEM images of the RuMoNi electrocatalyst after a durability test of 3,000 h at 500 mA cm^{-2}.

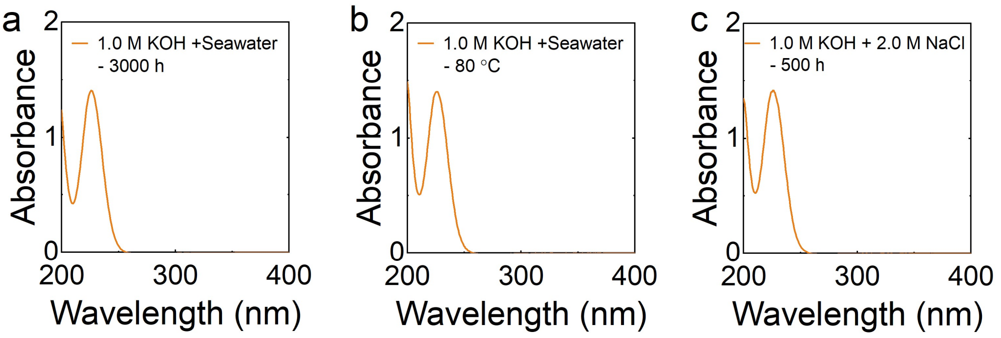

Supplementary Figure 22. UV-vis spectra of used electrolytes with the addition of 0.5 M KI aqueous solutions. **a)** 1.0 M KOH + seawater electrolyte tested for 3,000 h which is the electrolyte for the CP test in Fig. 3a. **b)** 1.0 M KOH + seawater electrolyte under 80 °C which is the electrolyte for the CP test in Fig. 3c. **c)** 1.0 M KOH + 2.0 M NaCl electrolyte tested for 500 h which is the electrolyte for the CP test in Fig. 3d.

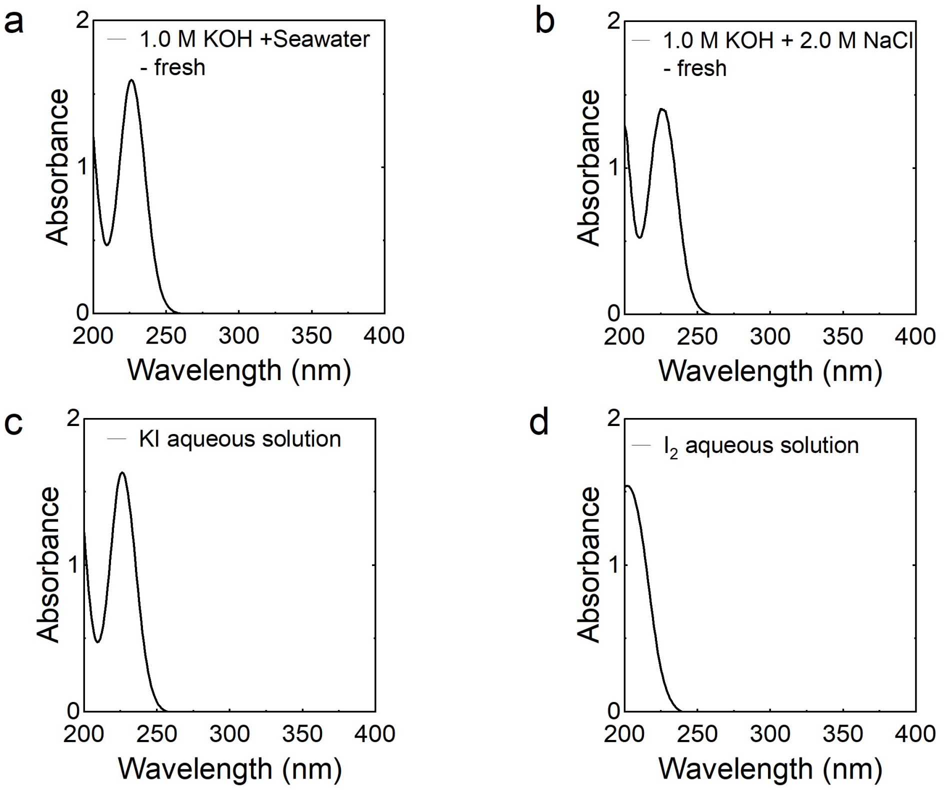

Supplementary Figure 23. UV-vis spectra of electrolytes and aqueous solutions. **a)** 1.0 M KOH + seawater electrolyte. **b)** 1.0 M KOH + 2.0 M NaCl electrolyte. **c)** KI aqueous solution. **d)** I_2 aqueous solution.

By comparing Supplementary Figures 22 and 23, we can see that the UV-vis spectra of the used electrolytes are the same as for the fresh electrolytes and KI aqueous solution. In addition, no obvious signal from I_2 was found. Therefore, these results indicate that the RuMoNi electrocatalyst retains its high OER selectivity in long-term operation.

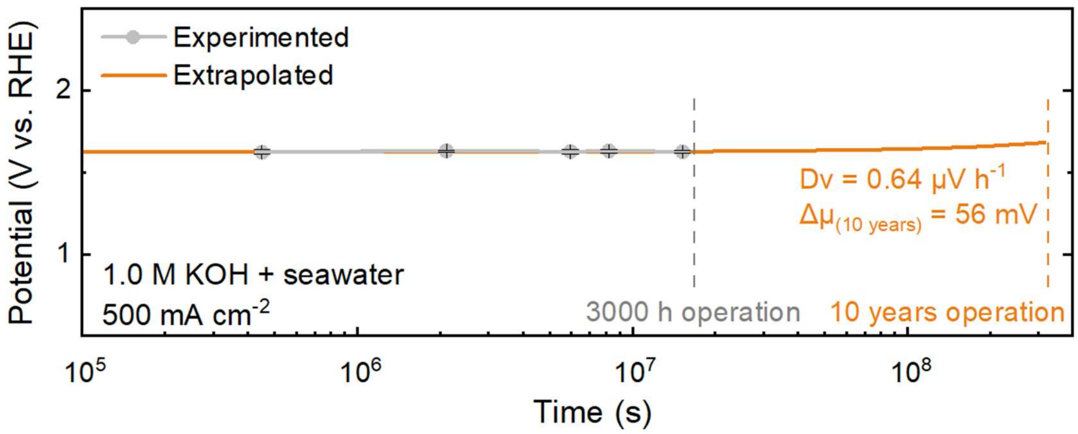

Supplementary Figure 24. Extrapolation of the durability test result of RuMoNi (500 mA cm^{-2}, 1.0 M KOH + seawater, 3,000 h, at 20 ± 2 °C). It is estimated that the RuMoNi electrocatalyst can operate well over 10 years with only a 56 mV degradation.

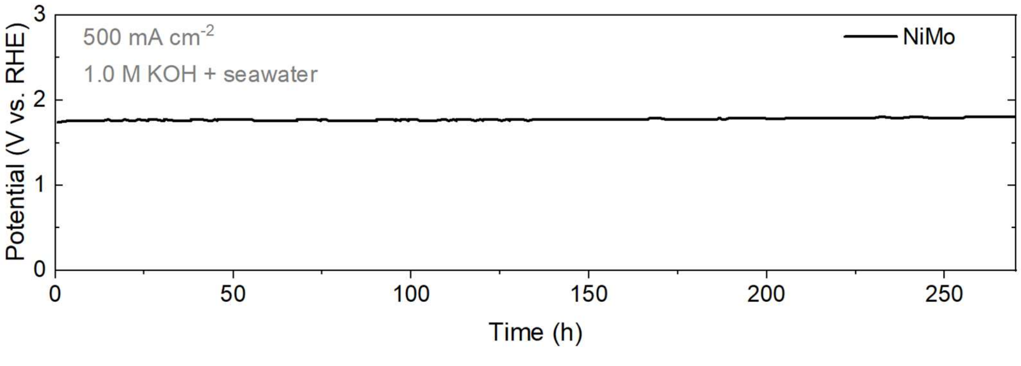

Supplementary Figure 25. Durability test of the NiMo catalyst for over 250 h recorded at a current density of 500 mA cm^{-2} in a 1.0 M KOH + seawater electrolyte at 20 ± 2 °C.

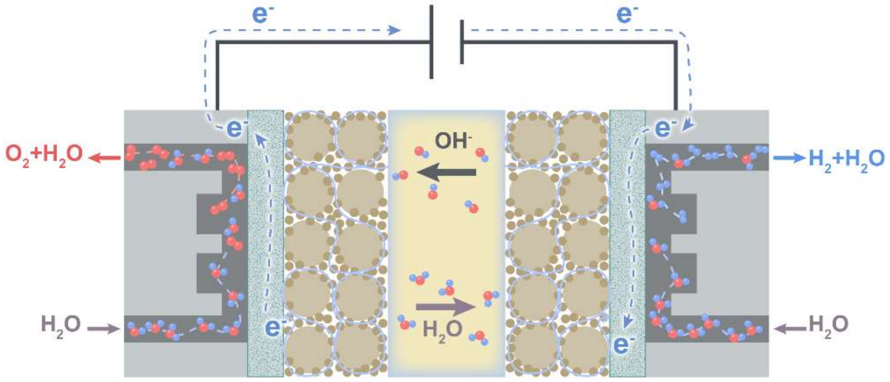

Supplementary Figure 26. Schematic of the configuration of the designed AEM electrolyzer. The left chamber is the anode chamber where the OER reaction happens, and the right chamber is the cathode chamber where the HER reaction happens. The yellow part in the middle is the anion exchange membrane. The brown parts beside the membrane are the catalyst layers.

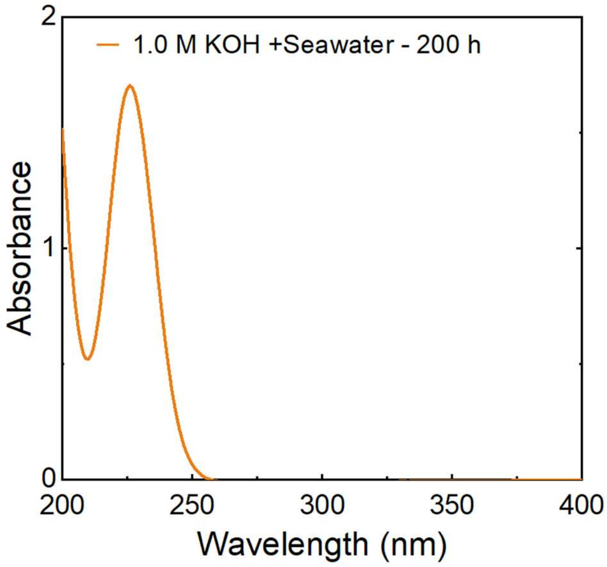

Supplementary Figure 27. UV-vis spectrum of used electrolytes after the durability test for the AEM electrolyzer with the addition of a 0.5 M KI aqueous solution.

Supplementary Table 1. Composition of seawater at 20 ± 2 ℃. The mass concentration of Cl^- and SO_4^{2-} were tested by ion chromatograph (IC). The mass concentration of Na^+, Mg^{2+}, and Ca^{2+} were tested by inductively coupled plasma optical emission spectroscopy (ICP-OES)

**Ions**	**Mass concentration (ppm)**	**Standard deviation (ppm)**
**Cl^-**	17683	288
**SO_4^{2-}**	2444	91
**Na^+**	9241	61
**Mg^{2+}**	1107	12
**Ca^{2+}**	361	3

Supplementary Table 2. Electrochemical analysis of various samples and their intrinsic activities for the OER in 1.0 M KOH + seawater electrolytes at 20 ± 2 ℃.

	C_{dl} (mF cm^{-1})	R_f	j @ 1.50 V vs. RHE (mA cm^{-2})	$j_{specific}$ @ 1.50 V vs. RHE (mA cm^{-2})
**RuMoNi**	4.3 ± 0.02	108 ± 0.1	51.7 ± 0.08	0.48 ± 0.001
**RuO_2**	14.6 ± 0.07	366 ± 0.8	6.1 ± 0.01	0.02 ± 0.001
**Ni foam**	1.1 ± 0.04	28 ± 0.5	0.9 ± 0.06	0.03 ± 0.004

Supplementary Table 3. The overpotentials of RuMoNi at 100, 500, and 1,000 mA cm^{-2} in different electrolytes at 20 °C.

**Current density**	**1M KOH (mV)**	**1M KOH + 0.5 M NaCl (mV)**	**1M KOH + Seawater (mV)**
**100 mA cm^{-2}**	290 ± 0.2	300 ± 0.3	291 ± 0.2
**500 mA cm^{-2}**	390 ± 0.1	400 ± 0.4	397 ± 0.4
**1,000 mA cm^{-2}**	480 ± 0.3	470 ± 0.3	484 ± 0.2

Supplementary Table 4. Fitting data of EIS data of RuMoNi, RuO_2, and Ni Foam at 20 °C.

**Catalyst**	**R_s (Ω)**	**R_c (Ω)**	**R_{ct} (Ω)**
**RuMoNi**	0.30 ± 0.005	0.14 ± 0.006	1.60 ± 0.004
**RuO_2**	0.27 ± 0.005	0.12 ± 0.004	5.54 ± 0.004
**Ni Foam**	0.21 ± 0.002	0.03 ± 0.005	24.72 ± 0.001

R_s is the resistance of the solution. R_c is the resistance of the corrosion layer and R_{ct} is the charge transfer resistance on the electrode surface. The charge transfer resistance of RuMoNi is much smaller than those of the RuO_2 and Ni Foam.

Supplementary Table 5. Dissolution rate of Ni from electrode to electrolyte during stability test at 500 mA cm^{-2} in 1.0 M KOH + seawater electrolyte at 20 °C.

**Time** (h)	**Ni foam** (mg/h)	**RuMoNi** (mg/h)	**Ratio** Ni foam to RuMoNi
**1**	2.7 ± 0.01	0.16 ± 0.003	16.9 ± 0.1
**2**	16.1 ± 0.01	0.03 ± 0.004	464.5 ± 0.1
**20**	-	~0	-
**100**	-	~0	-

Supplementary Table 6. A comparison of the current density and test duration of a CP test in an alkaline seawater electrolyte normalized by geometric area.

**Catalyst**	**Current density ($mA\ cm^{-2}$)**	**Duration (h)**	**Electrolyte**	**Reference**
Ni-MoN	500	100	1 M KOH seawater	[11]
NCMS/NiO	100	720	6 M KOH seawater	[12]
Ni_2P	500	40	1 M KOH seawater	[13]
NiMoN@NiFeN	500	100	1 M KOH seawater	[14]
NMN-NF	100	50	1 M KOH seawater	[15]
NiS Fe	400	1000	1 M KOH seawater	[16]
S-(Ni,Fe)OOH	100	100	1 M KOH seawater	[17]
NiIr-OOH	500	650	1 M KOH seawater	[18]
**RuMoNi**	500	3,000	1 M KOH seawater	This work

Supplementary Table 7. A comparison of D_V and D_A during a CP test in an alkaline seawater electrolyte normalized by geometric area.

Catalyst	D_V (μV h^{-1})	D_A (h^{-1})	Electrolyte	Reference
Ni-MoN	60.15	113.60	1 M KOH seawater	[11]
NCMS/NiO	3.19	12.02	6 M KOH seawater	[12]
Ni_2P	86.93	114.34	1 M KOH seawater	[13]
NiMoN@NiFeN	27.10	47.02	1 M KOH seawater	[14]
NMN-NF	21.68	67.12	1 M KOH seawater	[15]
NiS Fe	4.29	4.86	1 M KOH seawater	[16]
S-(Ni,Fe)OOH	11.77	39.40	1 M KOH seawater	[17]
NiIr-OOH	2.27	4.98	1 M KOH seawater	[18]
**RuMoNi**	0.64	0.039	1 M KOH seawater	This work

Supplementary Table 8. A comparison of the AEM seawater electrolyzer performance between RuMoNi and three other electrocatalysts.

Catalyst	Ni-doped FeOOH	NiFe-LDH	Fe, P-$NiSe_2$	**RuMoNi**
**Cell efficiency at 500 mA cm^{-2} (%)**	77.4	74.7	73.8	77.9
**Current density at 1.7 V (mA cm^{-2})**	729	175	815	877
**Highest current density (mA cm^{-2})**	800	1,000	1,144	2,000
**Current density of stability test (mA cm^{-2})**	500	200	800	500
**Time length of stability test (h)**	15	2	200	240
**Reference**	[19]	[20]	[21]	This work

Supplementary Note 1. Calculations of AEM electrolyzer efficiency and H_2 cost. These calculations only considered the electricity costs, based on the method proposed by literature[22,23].

Electrolyzer efficiency

H_2 production rate @ 0.5 A cm^{-2}

= (j A cm^{-2})(1 e$^-$/1.602 ×10^{-19} C)(1 H_2/2 e$^-$)

= 0.5 A cm^{-2} / (1.602 × 10^{-19} C x 2)

= 2.59 × 10^{-6} mol H_2 cm^{-2} s^{-1}

LHV of H_2

= 120 kJ g^{-1} H_2 = 2.42 × 10^{5} J mol^{-1} H_2

H_2 power out

= (2.59 × 10^{-6} mol cm^{-2} s^{-1}) × (2.42 × 10^{5} J mol^{-1})

= 0.627 W cm^{-2}

Electrolyzer Power of RuMoNi||RuMoNi

Electrolyzer Power (RuMoNi) @ 0.5 A cm^{-2}

= (0.5 A cm^{-2}) (1.61 V)

= 0.805 W cm^{-2}

Efficiency of RuMoNi||RuMoNi

= (H_2 Power Out) / (Electrolyzer Power)

= 0.627 W cm^{-2}/0.805 W cm^{-2}

= 77.9%

Efficiency of RuO_2||Pt/C

= (H_2 Power Out) / (Electrolyzer Power)

= 0.627 W cm^{-2}/1.097 W cm^{-2}

= 57.15%

Price per gasoline-gallon equivalent (GGE) H_2

= 1GGE H_2 / H_2 production rate × Electrolyzer power × Electricity bill

= 0.997 kg / (2.59 × 10^{-6} mol H_2.cm^{-2} s^{-1} × 2 kg/mol) × 0.805 W cm^{-2} × \$ 0.02 / kW h

= \$ 0.85 /GGE H_2

<< \$ 2 /GGE H_2 of U.S. Department of Energy (DOE) by 2026

Supplementary References

1. Ohyoshi, A., Götzfried, F. & Beck, W. Polynuclear carbonyl complexes of ruthenium and osmium with methylthiolate and bromide bridging ligands. *Chem. Lett.* **9**, 1537-1540 (1980).
2. Cimino, A. & Deangelis, B. A. Application of X-ray photoelectron-spectroscopy to study of molybdenum oxides and supported molybdenum oxide catalysts. *J. Catal.* **36**, 11-22 (1975).
3. Colton, R. J., Guzman, A. M. & Rabalais, J. W. Electrochromism in some thin-film transition-metal oxides characterized by X-ray electron spectroscopy. *J. Appl. Phys.* **49**, 409-416 (1978).
4. Fleisch, T. H. & Mains, G. J. An XPS study of the UV reduction and photochromism of MoO_3 and WO_3. *J. Chem. Phys.* **76**, 780-786 (1982).
5. Klaus, S., Cai, Y., Louie, M. W., Trotochaud, L. & Bell, A. T. Effects of Fe electrolyte impurities on $Ni(OH)_2$/NiOOH structure and oxygen evolution activity. *J. Phys. Chem. C* **119**, 7243-7254 (2015).
6. Kang, J. et al. Valence oscillation and dynamic active sites in monolayer NiCo hydroxides for water oxidation. *Nat. Catal.* **4**, 1050-1058 (2021).
7. Dieterle, M. & Mestl, G. Raman spectroscopy of molybdenum oxides. *Phys. Chem. Chem. Phys.* **4**, 822-826 (2002).
8. Xie, S., Chen, K., Bell, A. T. & Iglesia, E. Structural characterization of molybdenum oxide supported on zirconia. *J. Phys. Chem. B* **104**, 10059-10068 (2000).
9. Lee, S., Bai, L. & Hu, X. Deciphering iron-dependent activity in oxygen evolution catalyzed by nickel–iron layered double hydroxide. *Angew. Chem.* **132**, 8149-8154 (2020).
10. Wang, J. et al. Redirecting dynamic surface restructuring of a layered transition metal oxide catalyst for superior water oxidation. *Nat. Catal.* **4**, 212-222 (2021).
11. Wu, L. et al. Efficient alkaline water/seawater hydrogen evolution by a nanorod-nanoparticle-structured Ni-MoN catalyst with fast water-dissociation kinetics. *Adv. Mater.* **34**, 2201774 (2022).
12. Seenivasan, S. & Kim, D.-H. Engineering the surface anatomy of an industrially durable $NiCo_2S_4$/$NiMo_2S_4$/NiO bifunctional electrode for alkaline seawater electrolysis. *J. Mater. Chem. A* **10**, 9547-9564 (2022).
13. Wu, L. et al. Heterogeneous bimetallic phosphide Ni_2P-Fe_2P as an efficient bifunctional catalyst for water/seawater splitting. *Adv. Funct. Mater.* **31**, 2006484 (2020).
14. Yu, L. et al. Non-noble metal-nitride based electrocatalysts for high-performance alkaline seawater electrolysis. *Nat. Commun.* **10**, 5106 (2019).
15. Li, Y. et al. Sandwich structured Ni_3S_2-MoS_2-Ni_3S_2@Ni foam electrode as a stable bifunctional electrocatalyst for highly sustained overall seawater splitting. *Electrochim. Acta* **390**, 138833 (2021).
16. Kuang, Y. et al. Solar-driven, highly sustained splitting of seawater into hydrogen and oxygen fuels. *Proc. Natl. Acad. Sci. U. S. A.* **116**, 6624-6629 (2019).
17. Yu, L. et al. Ultrafast room-temperature synthesis of porous S-doped Ni/Fe (oxy)hydroxide electrodes for oxygen evolution catalysis in seawater splitting. *Energy Environ. Sci.* **13**, 3439-3446 (2020).
18. You, H. et al. Monolayer NiIr-layered double hydroxide as a long-lived efficient oxygen evolution catalyst for seawater splitting. *J. Am. Chem. Soc.* **144**, 9254-9263 (2022).
19. Park, Y. S. et al. High-performance anion exchange membrane alkaline seawater electrolysis. *J.*

Mater. Chem. A **9**, 9586-9592 (2021).

20. Dresp, S. et al. Efficient direct seawater electrolysers using selective alkaline NiFe-LDH as OER catalyst in asymmetric electrolyte feeds. *Energy Environ. Sci.* **13**, 1725-1729 (2020).
21. Chang, J. et al. Dual-doping and synergism toward high-performance seawater electrolysis. *Adv. Mater.* **33**, 2101425 (2021).
22. Hao, S. et al. Torsion strained iridium oxide for efficient acidic water oxidation in proton exchange membrane electrolyzers. *Nat. Nanotechnol.* **16**, 1371-1377 (2021).
23. King, L. A. et al. A non-precious metal hydrogen catalyst in a commercial polymer electrolyte membrane electrolyser. *Nat. Nanotechnol.* **14**, 1071-1074 (2019).
